# Interleukin-1β Alters Hebbian Synaptic Plasticity in Multiple Sclerosis

**DOI:** 10.3390/ijms21196982

**Published:** 2020-09-23

**Authors:** Mario Stampanoni Bassi, Fabio Buttari, Carolina Gabri Nicoletti, Francesco Mori, Luana Gilio, Ilaria Simonelli, Nicla De Paolis, Girolama Alessandra Marfia, Roberto Furlan, Annamaria Finardi, Diego Centonze, Ennio Iezzi

**Affiliations:** 1 Unit of Neurology & Neurorehabilitation, IRCCS Neuromed, 86077 Pozzilli (IS), Italy; m.stampanonibassi@gmail.com (M.S.B.); fabio.buttari@gmail.com (F.B.); gilio.luana@gmail.com (L.G.); depaolisnicla@gmail.com (N.D.P.); marfia@med.uniroma2.it (G.A.M.); ennio.iezzi@neuromed.it (E.I.); 2 Multiple Sclerosis Clinical and Research Unit, Department of Systems Medicine, University of Rome Tor Vergata, 00133 Rome, Italy; carolgabri@gmail.com (C.G.N.); francesco.mori@uniroma2.it (F.M.); 3 Service of Medical Statistics & Information Technology, Fondazione Fatebenefratelli per la Ricerca e la Formazione Sanitaria e Sociale, 00153 Rome, Italy; ilaria.simonelli@afar.it; 4 Clinical Neuroimmunology Unit, Institute of Experimental Neurology, Division of Neuroscience, San Raffaele Scientific Institute, 20132 Milano, Italy; furlan.roberto@hsr.it (R.F.); finardi.annamaria@hsr.it (A.F.)

**Keywords:** paired associative stimulation (PAS), multiple sclerosis (MS), interleukin (IL)-1β, synaptic plasticity, long-term potentiation (LTP), transcranial magnetic stimulation (TMS)

## Abstract

In multiple sclerosis (MS), inflammation alters synaptic transmission and plasticity, negatively influencing the disease course. In the present study, we aimed to explore the influence of the proinflammatory cytokine IL-1β on peculiar features of associative Hebbian synaptic plasticity, such as input specificity, using the paired associative stimulation (PAS). In 33 relapsing remitting-MS patients and 15 healthy controls, PAS was performed on the abductor pollicis brevis (APB) muscle. The effects over the motor hot spot of the APB and abductor digiti minimi (ADM) muscles were tested immediately after PAS and 15 and 30 min later. Intracortical excitability was tested with paired-pulse transcranial magnetic stimulation (TMS). The cerebrospinal fluid (CSF) levels of IL-1β were calculated. In MS patients, PAS failed to induce long-term potentiation (LTP)-like effects in the APB muscle and elicited a paradoxical motor-evoked potential (MEP) increase in the ADM. IL-1β levels were negatively correlated with the LTP-like response in the APB muscle. Moreover, IL-1β levels were associated with synaptic hyperexcitability tested with paired-pulse TMS. Synaptic hyperexcitability caused by IL-1β may critically contribute to alter Hebbian plasticity in MS, inducing a loss of topographic specificity.

## 1. Introduction

Multiple sclerosis (MS) is an immune-mediated disease characterized by inflammation of the central nervous system (CNS), which is associated with demyelinating white matter lesions and neurodegeneration. Relapsing-remitting (RR)-MS is characterized by a very variable disease course, with stable phases alternating with acute relapses. The ability to compensate for ongoing brain damage critically influences the disease course, promoting clinical stability and preventing the accumulation of disability. Synaptic plasticity, and particularly long-term potentiation (LTP), is one of the main physiological mechanisms involved in clinical recovery after brain damage [1,2]. Accordingly, it has been speculated that efficient LTP induction could have a positive influence on MS disease course [3].

Studies in experimental autoimmune encephalomyelitis (EAE), an animal model of MS, and in patients with MS, have shown that CNS inflammation affects synaptic functioning. In particular, specific proinflammatory mediators released by the immune cells influence synaptic transmission and plasticity. Interleukin (IL)-1β is one of the most important proinflammatory cytokines associated with MS pathogenesis [4] and plays a crucial role in regulating neuronal functioning in both physiological and pathological conditions [5,6,7]. Experimental studies in EAE have demonstrated that IL-1β alters synaptic functioning, promoting synaptic hyperexcitability and glutamate-mediated excitotoxicity [8,9]. Accordingly, raised IL-1β signaling has been associated with worse disease course and increased neurodegeneration in EAE and in patients with MS [10,11].

However, how IL-1β modifies LTP expression has not yet been conclusively clarified. In fact, studies in mice with EAE have demonstrated both impaired hippocampal LTP [12] and pathologically increased LTP [13] in response to IL-1β. Notably, it has been demonstrated that IL-1β induces both increased glutamatergic transmission and defective GABAergic signaling [8,9,14]. Altered LTP induction has also been found in patients with MS using transcranial magnetic stimulation (TMS). In particular, in RR-MS, a paradoxical LTP-like effect has been evidenced in response to continuous theta burst stimulation (cTBS), a protocol inducing long-term depression (LTD), and has been correlated with IL-1β cerebrospinal fluid (CSF) levels [15].

In the present study, to better characterize how IL-1β could affect LTP in human MS, we investigated in a group of RR-MS patients the effect of IL-1β on LTP induction using paired associative stimulation (PAS). This TMS protocol is particularly suitable to investigate the peculiar features of associative Hebbian synaptic plasticity, such as input specificity, which can be particularly altered in MS patients due to the imbalance between excitatory and inhibitory synaptic transmission induced by neuroinflammation.

## 2. Results

The clinical characteristics of MS patients and healthy controls are shown in Table 1. TMS was well tolerated by all participants, and no adverse effects were reported.

### 2.1. PAS-Induced LTP-like Plasticity is Altered in RR-MS Patients

Repeated measures analysis of variance (RM-ANOVA) exploring the effects of PAS in MS patients and controls showed a significant MEP size increase after PAS (time effect: F = 12.336; *p* < 0.001), and the effect differed between abductor pollicis brevis (APB) and abductor digiti minimi (ADM) muscles (muscle effect: F = 11.319; *p* = 0.002). The MEP increase after PAS was significantly greater in controls compared with MS patients (interactions time × group effect: F = 3.557; *p* = 0.022) and more evident in APB compared to ADM muscle (interaction time × muscle effect: F = 3.979; *p* = 0.014). Finally, significant differences were found when comparing the PAS response in APB and ADM muscles between controls and MS patients (interaction muscle × group effect: F = 44.854; *p* < 0.001; interaction time × muscle × group effect: F = 15.061; *p* < 0.001).

**Figure 1:**
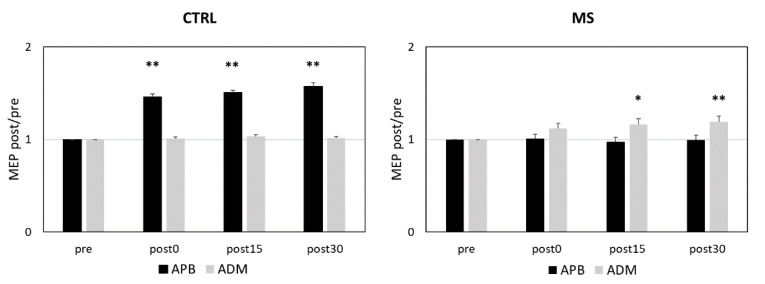
PAS Effects in RR-MS Patients and Controls. Figure 1 legend. In healthy subjects, PAS elicited the expected homosynaptic LTP-like effect in the APB muscle (**left panel**), whereas in MS patients, PAS elicited heterosynaptic LTP in the ADM muscle (**right panel**). * *p* ≤ 0.05; ** *p* ≤0.01. The *p* values refer to the comparisons between pre and post 0, post 15, and post 30 in each muscle. All *p* values were adjusted by Benjamini–Hochberg correction. Abbreviations: APB (abductor pollicis brevis); ADM (abductor digiti minimi); MEP (motor-evoked potential); PAS (paired associative stimulation); RR-MS (relapsing-remitting multiple sclerosis).

Post hoc analyses showed that in control subjects, PAS induced a significant LTP-like effect in the APB muscle and had no effect in the ADM muscle. Conversely, MS patients lacked the expected LTP-like effect in the APB and showed a paradoxical motor-evoked potential (MEP) increase in the ADM (Figure 1).

No significant correlations emerged between PAS effect and the clinical and demographic characteristics explored (age, disease duration, disability; all *p* > 0.2).

### 2.2. CSF IL-1β Alters PAS Effects

To explore the influence of IL-1β on synaptic plasticity in MS patients, we analyzed the correlation between the CSF levels of this cytokine and the PAS-induced LTP-like effect.

**Figure 2:**
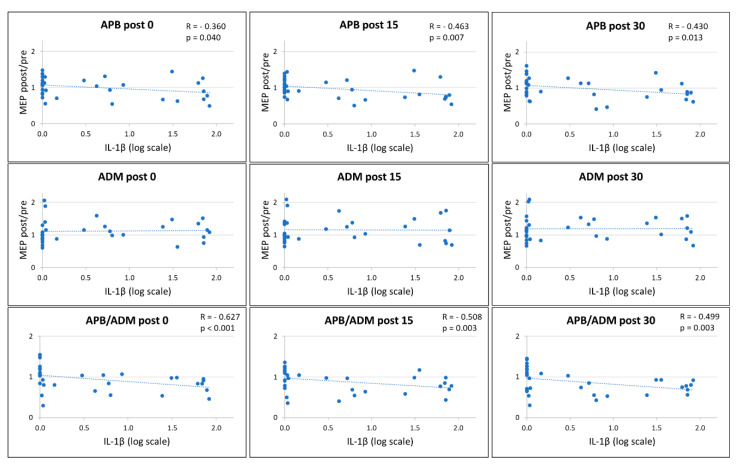
Correlation between IL-1β CSF Levels and PAS Effects. Figure 2 legend. A negative correlation was found between IL-1β CSF concentrations and LTP-like effects in the APB muscle at post 0, post 15, and post 30 (**upper panels**); conversely, no significant correlation emerged with LTP-like effects in the ADM muscle (**middle panels**). A significant negative correlation was found between IL-1β CSF levels and the ratio of the LTP-like effect induced in APB and ADM muscles (**lower panels**). Abbreviations: APB (abductor pollicis brevis); ADM (abductor digiti minimi); CSF (cerebrospinal fluid); IL (interleukin); MEP (motor-evoked potential).

A significant negative correlation emerged between IL-1β CSF concentrations and the amount of LTP-like effect induced by the PAS protocol in the APB muscle at post 0 (Spearman’s r (33) = −0.360, *p* = 0.040, B-H adjusted *p* = 0.040) post 15 (Spearman’s r (33) = −0.463, *p* = 0.007 B-H adjusted *p* = 0.011), and post 30 (Spearman’s r (33) = −0.430, *p* = 0.013, B-H adjusted *p* = 0.017) (Figure 2). Conversely, no significant correlations emerged between the CSF levels of this cytokine and the effect of PAS explored on the ADM.

Finally, a significant negative correlation emerged between IL-1β CSF concentrations and the ratio of the LTP-like effect induced by the PAS protocol on APB and ADM muscles at post 0 (Spearman’s r (33) = −0.627, *p* < 0.001, B-H adjusted *p* < 0.001), post 15 (Spearman’s r (33) = −0.508, *p* = 0.003, B-H adjusted *p* = 0.006), and post 30 (Spearman’s r (33) = −0.499, *p* = 0.003, B-H adjusted *p* = 0.006).

### 2.3. Detectable IL-1β CSF Levels are Associated with Paradoxical Response to PAS

To further explore the impact of IL-1β CSF concentrations on PAS, we divided MS patients into two groups according to the presence of IL-1β in the CSF. Patients with detectable IL-1β in the CSF (IL-1β positive) amounted to 20, while IL-1β was undetectable in the CSF of 13 patients (IL-1β negative). The clinical characteristics of the two groups are shown in Table 2. 

**Figure 3:**
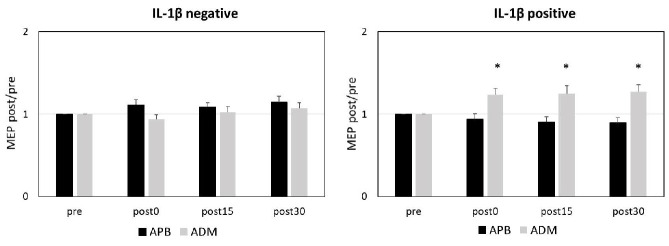
IL-1β CSF Detectability and PAS Effects. Figure 3 legend. In the IL-1β negative group, PAS failed to induce LTP-like effects in both APB and ADM muscles (**left panel**), whereas in the IL-1β positive group, PAS elicited an abnormal LTP in the ADM muscle (**right panel**). * *p* ≤ 0.05. The *p* values refer to the comparisons between pre and post 0, post 15, and post 30 in each muscle. All *p* values were adjusted by Benjamini–Hochberg correction. Abbreviations: APB (abductor pollicis brevis); ADM (abductor digiti minimi); CSF (cerebrospinal fluid); IL (interleukin); PAS (paired associative stimulation).

In MS patients, RM-ANOVA showed that response to PAS in APB and ADM muscles significantly differed according to the presence of IL-1β in the CSF (interactions muscle x group: F = 17.162; *p* < 0.001; interaction time x muscle x group effect: F = 6.144; *p* = 0.002). Post hoc comparisons showed in the IL-1β positive group a significant LTP-like effect in the ADM muscle but not in the APB, while no significant LTP-like effect was observed in the IL-1β negative group on both muscles (Figure 3).

### 2.4. IL-1β and Intracortical Excitability

**Figure 4:**
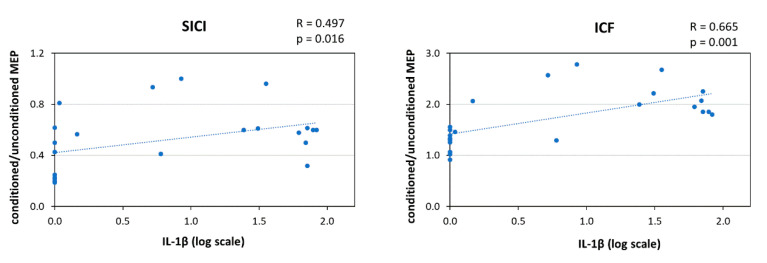
Correlation between IL-1β CSF Levels and Inhibitory/Excitatory Intracortical Transmission. Figure 4 legend. IL-1β CSF levels positively correlated with reduced intracortical inhibition (**left panel**) and increased intracortical facilitation (**right panel**). Abbreviations: CSF (cerebrospinal fluid); ICF (intracortical facilitation); IL (interleukin); MEP (motor-evoked potential); SICI (short-interval intracortical inhibition).

To explore whether IL-1β affects intracortical excitability, we correlated the CSF concentration of this cytokine with the short-interval intracortical inhibition (SICI) and intracortical facilitation (ICF). A significant correlation emerged between IL-1β and both SICI (Spearman’s r (23) = 0.497, *p* = 0.016, B-H adjusted *p* = 0.018) and ICF (Spearman’s r (23) = 0.665, *p* = 0.001, B-H adjusted *p* = 0.004) (Figure 4). SICI and ICF did not correlate with the PAS-induced effect on the ADM muscle. Finally, no significant correlations were found between SICI, ICF, and the clinical and demographic characteristics (age, disease duration, disability, and disease activity; all *p* > 0.2).

## 3. Discussion

The PAS protocol significantly changes cortical excitability by combining repeated TMS activation of M1 with peripheral nerve stimulation, separated by specific time intervals [16,17]. This protocol resembles the Hebbian spike-timing-dependent plasticity described in animal experiments where pre- and post-synaptic neuronal activation induces changes in synaptic efficacy [18,19]. Repeated low-frequency electrical stimulation of the median nerve followed 25 ms after by single-pulse TMS over the cortical representation of the contralateral APB muscle is able to increase the amplitude of the MEPs in the APB muscle, persisting up to 60 min. The effect of PAS requires the activity of the N-Methyl-D-Aspartate (NMDA) receptor as shown by the administration of NMDA antagonists [20]. Moreover, PAS effects are bidirectional and strongly governed by temporal rules, as LTD is induced when the TMS pulse follows the electric nerve stimulation at an ISI of 10 ms [21]. Finally, PAS effects are somatotopically specific, being evident only in the muscle receiving homotopical input by afferent stimulation and TMS [20,22]. Therefore, associativity and input specificity of PAS follow the Hebbian rules of synaptic plasticity. 

In the present study we found that CSF IL-1β expression is associated with altered PAS25-induced LTP in RR-MS patients. Patients with detectable IL-1β showed both absent LTP in the APB muscle and abnormal LTP expression in the ADM muscle. These results suggest that IL-1β may promote a profound alteration of Hebbian synaptic plasticity mechanisms, characterized by impaired homosynaptic LTP expression and loss of topographic specificity leading to heterosynaptic effects. 

The finding that IL-1β CSF detectability is associated with an absent LTP-like effect is in line with previous studies showing that inflammation in MS is associated with altered LTP induction [23,24]. TMS studies investigating the homosynaptic effects of PAS25 in MS patients have shown that elevated CSF levels of the proinflammatory cytokine IL-6 negatively correlated with the LTP-like effects induced by the PAS protocol [24]. Conversely, comparable effects of PAS25 between remitting MS patients and controls have been reported [25].

Notably, inflammation has also been associated with a paradoxical LTP-like response produced by LTD-inducing protocols both in EAE and MS [13,15,26]. IL-1β has been specifically associated with LTP induction in response to low frequency stimulation in mice hippocampal slices [13] and to LTP-like response after cTBS [15]. Lacking LTD-like effects during MS relapses may depend on reduced GABAergic transmission [10,27]. Accordingly, IL-1β has been associated with synaptic hyperexcitability due to enhanced glutamatergic and impaired GABAergic transmission in both EAE and MS, promoting excitotoxic neurodegeneration [10,14]. Interestingly, blocking IL-1β signaling with an IL-1 receptor antagonist normalized synaptic transmission and plasticity [10,15], confirming the specific role of IL-1β as a key player of inflammatory synaptopathy in EAE and MS. While the increased IL-1β levels could explain the lack of PAS-induced homosynaptic LTP, the altered balance between excitatory and inhibitory transmission caused by IL-1β might have altered the topographic specificity of PAS in RR-MS responsible for abnormal heterosynaptic LTP shown here.

Due to the positive-feedback nature of LTP, uncontrolled increases of synaptic efficacy can destabilize neuronal activity, and therefore homeostatic mechanisms are required to constrain synaptic strength in a dynamic physiological range [28]. A compensatory mechanism aimed at preventing excessive synaptic plasticity from developing relies on the Bienenstock–Cooper–Munro theory [29], postulating that the threshold for LTP induction varies as a function of the integrated postsynaptic activity. Accordingly, low levels of postsynaptic activity favour LTP induction whereas high levels block it. Another mechanism involved in the homeostatic control of synaptic activity is synaptic scaling. This form of homeostatic plasticity modifies the expression of α-amino-3-hydroxy-5-methyl-4-isoxazolepropionic acid receptors (AMPARs), inducing increases or decreases of neuronal excitability (upscaling and downscaling, respectively). Unlike Hebbian plasticity, scaling has a negative-feedback nature and lacks input specificity as it involves all synapses in a neuron [30,31]. This mechanism contributes to counterbalance the synaptic instability produced by unrestrained increases in synaptic excitability induced by LTP alone.

Altered spatial specificity of the PAS protocol has been previously reported in other neurological manifestations, in particular in patients with dystonia [22,32], and has been related to a defective neuronal inhibition [33]. 

Altered mechanisms of homeostatic control of synaptic plasticity have also been demonstrated in EAE and MS. Experimental studies have demonstrated that inflammatory molecules influence the induction and maintenance of synaptic scaling in the brain [34,35,36,37]. Accordingly, in EAE, increased post-synaptic glutamatergic excitatory currents have been evidenced before the appearance of clinical manifestations [8]. These alterations have been associated with exacerbated neuronal damage, which can be prevented by the administration of AMPA blockers [8].

In the present study we found that IL-1β CSF levels correlated with enhanced ICF and reduced SICI in RR-MS patients. Therefore, heterosynaptic LTP-like effects on the ADM muscle elicited by PAS might depend on synaptic hyperexcitability associated with raised IL-1β CSF concentrations.

Given the pivotal role of IL-1β in the pathogenesis of MS and in the development of synaptic alterations, anti-IL-1β drugs might have a potential therapeutic role. Although recent reports suggest a possible beneficial effect of anti-IL-1β therapies (i.e., anakinra and canakinumab) in MS [38], current evidence is limited. A clinical trial is ongoing to test the efficacy and safety of anakinra in patients with MS (ClinicalTrials.gov: NCT04025554).

Although various proinflammatory and anti-inflammatory molecules can regulate synaptic plasticity in MS [39], we focused on the proinflammatory cytokine IL-1β which has been previously identified as one of the main determinants of the inflammatory synaptopathy in MS [40]. However, a wider set of CSF molecules should be analyzed to better explore the impact of the inflammatory milieu on synaptic functioning, and additional preclinical investigations are needed to clarify the pathophysiological mechanisms of IL-1β-driven synaptic alterations. Related to this issue, another important feature to address is the contribution of different disease phases in synaptic alterations in MS. In this study, we did not find a significant difference in PAS response between relapsing and remitting patients; however, the presence of radiological activity has been previously associated with synaptic alterations [23,26] and may represent a possible confounding factor.

PAS is particularly suitable for investigating some properties of spike timing-dependent plasticity in humans. Our results have shown that MS synaptic hyperexcitability induced by IL-1β may critically contribute to alter Hebbian plasticity, inducing a loss of topographic specificity.

## 4. Materials and Methods

### 4.1. MS Patients

The study involving human subjects was approved by the Ethics Committee of the University Tor Vergata Hospital in Rome, Italy (approval code 123/15, approval date 25 September 2015). All patients gave written informed consent. A group of 33 RR-MS patients admitted to the neurological clinic of University Tor Vergata Hospital participated in the study. MS diagnosis was based clinical, laboratory, and MRI parameters [41]. A group of 15 healthy controls was also included.

All patients underwent clinical examination, MRI scan, CSF withdrawal, and TMS evaluation during hospitalization. Corticosteroids or disease modifying therapies were initiated later if indicated. All patients were asymptomatic in the upper right limb. Disability was assessed using the Expanded Disability Status Scale (EDSS) [42]. Disease duration was calculated as the number of months from disease onset to the time of diagnosis. MRI examination consisted of dual-echo proton density, fast fluid-attenuated inversion recovery, T2-weighted spin-echo images, and pre-contrast and post-contrast T1-weighted spin-echo images. 

### 4.2. CSF Collection and Analysis

After lumbar puncture, CSF was centrifuged and immediately stored at −80 °C until analysed using a Bio-Plex multiplex cytokine assay (Bio-Rad Laboratories, Hercules, CA, USA) according to the manufacturer’s instructions. CSF concentrations of IL-1β were calculated according to a standard curve generated for the specific target and expressed as picograms per milliliter (pg/mL). 

### 4.3. Transcranial Magnetic Stimulation

MEPs were elicited through a figure-of-eight coil with an external loop diameter of 70 mm connected to a Magstim 200^2^ magnetic stimulator (The Magstim Company Ltd., Whitland, Dyfed, UK). The coil was held tangentially to the scalp with the handle pointing backward and away from the midline at about 45°, in the optimal scalp sites (hot spots) to evoke MEPs in the contralateral APB and ADM muscles. Raw electromyographic signals were recorded with surface electrodes. Responses, sampled at 5 KHz with a CED 1401 A/D laboratory interface (Cambridge Electronic Design, Cambridge, UK), were amplified and filtered (bandpass 20 Hz to 2 kHz) with a Digitimer D360 amplifier (Digitimer Ltd., Welwyn Garden City, Hertfordshire, UK), then recorded by a computer with Signal software (Cambridge Electronic Design).

Resting motor threshold (RMT) was defined as the lowest stimulus intensity able to evoke MEPs at rest with peak-to-peak amplitude of 50 µV in five out of ten consecutive trials. Active motor threshold (AMT) was defined as the lowest intensity able to elicit MEPs of 100 uV in five out of ten consecutive stimuli, during a slight voluntary contraction of the target muscle.

Intracortical excitability was assessed by testing the SICI and ICF with a paired-pulse TMS paradigm. A conditioning stimulus (CS) set at an intensity of 80% AMT was delivered before a test stimulus (TS) with an intensity set to obtain MEPs with peak-to-peak amplitude of 0.5–1.0 mV [43,44]. For SICI, the interstimulus interval (ISI) elapsing between the CS and the TS was 3 ms, whereas for ICF the ISI was 10 ms. Three different conditions (10 test pulses given alone and 10 conditioned pulses for each ISI) were randomly tested. The conditioned MEPs were expressed as a percentage of the unconditioned MEPs.

LTP-like cortical plasticity was explored with the PAS protocol [16]. Median nerve electric shocks were followed by single TMS pulses over the abductor pollicis brevis (APB) muscle hot spot with an ISI of 25 ms. The median nerve was stimulated at the wrist with a constant current stimulator (model DS7A, Digitimer Ltd.) through a pair of surface electrodes (0.2 ms duration, cathode proximal). The intensity of the TMS pulses was set to evoke MEPs of about 0.5–1 mV peak-to-peak amplitude in the APB and in the ADM muscles at baseline. The same intensity was used to elicit MEPs after PAS. The median nerve was stimulated with an intensity set at 300% of the perceptual threshold. Two hundred pairs of electric and magnetic stimuli were repetitively delivered at a rate of 0.25 Hz. Twenty MEPs were recorded from the APB and from the ADM muscles at the baseline, immediately after PAS (post 0) and 15 and 30 min later (post 15 and post 30). At each time interval after PAS, MEP amplitudes were averaged and normalized to the mean baseline amplitude.

### 4.4. Statistical Analysis

Normality distribution of continuous variables was assessed by Shapiro–Wilk test. Data were expressed as the mean (standard deviation, SD) or, when necessary, the median (interquartile range, IQR). Categorical variables were shown as absolute (n) and relative frequency (%). Logarithmic transformation was applied to reduce the skewness of data distribution and better approximate the normal distribution. Pearson’s correlation or, if data were not normally distributed, Spearman’s non-parametric correlation, was applied to evaluate possible associations between continuous variables. The relationship between two continuous variables was depicted by a scatter plot. 

Possible differences in PAS response between patients and controls were explored with RM-ANOVA with time (pre, post 0, post 15, post 30) and muscle (APB, ADM) as within-subject factors and group (SM, Controls) as between-subject factors. Only for the MS sample was an RM-ANOVA with time (pre, post 0, post 15, post 30) and muscle (APB, ADM) as within-subject factors and MS group (MS IL1-β positive, MS IL-β negative) as between-subject factors performed to assess whether IL-1β CSF detectability influenced the PAS response. For post-hoc comparisons, two-tailed paired sample t tests were conducted to compare pre- vs post- (0, 15, 30) MEP amplitudes. For multiple comparisons, Benjamini–Hochberg correction was applied.

## Figures and Tables

**Table 1:** Clinical Characteristics of MS Patients and Controls.

		MS (33)	Controls (15)
Sex, females	N (%)	19 (57.6%)	10 (66.7%)
Age, years	mean (SD)	35.51 (9.34)	28.9 (7.4)
Disease duration, months	median (IQR)	13 (8–29)	-
Radiological activity	N (%)	13 (39.4%)	-
EDSS	median (IQR)	1.5 (1–2)	-
IL-1β, pg/mL	median (IQR)	0.1 (0–26.67)	-

Abbreviations: EDSS (expanded disability status scale); IL (interleukin); IQR (interquartile range); MS (multiple sclerosis); SD (standard deviation).

**Table 2:** Clinical Characteristics of MS Patients According to IL-1β Group.

		IL-1β Negative (13)	IL-1β Positive (20)
Sex, females	N (%)	8 (61.5%)	11 (50%)
Age, years	mean (SD)	38 (11.72)	33.9 (7.28)
Disease duration, months	median (IQR)	12 (9–35)	14 (6.5–31.5)
Radiological activity	N (%)	4 (30.8%)	9 (45%)
EDSS	median (IQR)	1.5 (1–2)	1.75 (1–2)

Abbreviations: EDSS (expanded disability status scale); IL (interleukin); IQR (interquartile range); MS (multiple sclerosis); SD (standard deviation).

## Abbreviations

ADM	abductor digiti minimi
AMPA	α-amino-3-hydroxy-5-methyl-4-isoxazolepropionic acid
AMT	active motor threshold
APB	abductor pollicis brevis
CS	conditioning stimulus
cTBS	continuous theta burst stimulation
EDSS	expanded disability status scale
ICF	intracortical facilitation
IL	interleukin
IQR	interquartile range
ISI	interstimulus interval
iTBS	intermittent theta burst stimulation
LTD	long-term depression
LTP	long-term potentiation
MEP	motor-evoked potential
NMDA	N-Methyl-D-Aspartate
PAS	paired associative stimulation
RMT	resting motor threshold
RR	relapsing–remitting
SD	standard deviation
SICI	short-interval intracortical inhibition
TMS	transcranial magnetic stimulation
TS	test stimulus
